# Impact of embryonic manipulations on core body temperature dynamics and survival in broilers exposed to cyclic heat stress

**DOI:** 10.1038/s41598-022-19063-1

**Published:** 2022-09-06

**Authors:** Chris Major Ncho, Akshat Goel, Vaishali Gupta, Chae-Mi Jeong, Yang-Ho Choi

**Affiliations:** 1 Department of Animal Science, Gyeongsang National University, Jinju, 52828 Republic of Korea; 2 Division of Applied Life Sciences (BK21 Plus Program), Gyeongsang National University, Jinju, 52828 Republic of Korea; 3 Institute of Agriculture and Life Sciences, Gyeongsang National University, Jinju, 52828 Republic of Korea

**Keywords:** Animal behaviour, Animal physiology

## Abstract

Ambient temperature-associated stress has been shown to affect the normal physiological functions of birds. The recent literature indicated that both, embryonic thermal manipulation (ETM) and in ovo feeding (IOF) of γ-aminobutyric acid (GABA) can mitigate the deleterious effects of heat stress (HS) in young broiler chicks. Therefore, this study intended to assess the effects of cyclic HS (32 ± 1 °C, 4 h/day from day 29 to 35) on rectal temperatures (RTs) and survival in broiler chickens after ETM and in IOF of GABA. A total of 275 RT data points and survival data were collected from chicks assigned to the following five treatments: chicks hatched from control eggs (CON); chicks hatched from control eggs but exposed to HS (CON + HS); chicks hatched from eggs injected at 17.5 days of incubation with 0.6 mL of 10% GABA and exposed to HS (G10 + HS); chicks hatched from thermally manipulated eggs (39.6 °C, 6 h/day from embryonic days 10 to 18) and exposed to HS (TM + HS); chicks hatched from eggs that received both previous treatments during incubation and exposed to HS (G10 + TM + HS). Under thermoneutral conditions, RTs increased quadratically from 39.9 ± 0.2 °C at hatching to 41.4 ± 0.1 °C at 8 days of age. When exposed to cyclic HS during the last week of rearing, the birds’ RTs tended to decrease at the end of the heat stress challenge (from 43.0 ± 0.2 °C on day 29 to 42.4 ± 0.1 °C on day 35). A stepwise Cox regression indicated that treatment was predictive of birds’ survival. Hazard ratios (HR) and their confidence intervals (CI) were calculated to assess the likelihood of death during the trial. The birds, belonging to the G10 + TM + HS group, were less likely to die under HS (HR 0.11, 95% CI 0.02 to 0.91, P = 0.041) compared to the CON + HS birds. Taken together, the combination of ETM and GABA IOF may help mitigate the drawbacks of cyclic HS by improving the survival of broilers.

## Introduction

Worldwide, poultry production efficiency is negatively impacted by the overall increase in ambient temperature. The rise in both temperature and humidity above the comfort zone usually causes heat stress (HS). High rearing temperatures lead to abnormal growth rate, feed consumption, feed efficiency, and increased mortality^1^. Moreover, other significant drawbacks of HS such as oxidative stress, disruption in lipid metabolism, and gut integrity alteration have been highlighted in recent studies^2^.

Avian species, like the majority of mammals, can maintain their core body temperature within the same range when the ambient temperature fluctuates^3^. Like other homeotherms, birds have developed a physical and metabolic mechanism for thermoregulation^4^. Adults chickens have an average body temperature ranging from 41 to 42 °C under thermoneutral conditions^5^. However, when the temperature rises to a certain extent, broilers’ ability to dissipate heat is markedly diminished. Subsequently, their body temperature rises, and chickens start to drink water, pant, and/or spread their wings.

Many solutions have been proposed for mitigating the deleterious effects of HS in broiler production. Among them are ventilation systems, water management, genetic modification, and dietary modulation^6^. γ-aminobutyric acid (GABA) has been showing promising results^7–9^. GABA, as one of the main inhibitory neurotransmitters in the brain, has the function to decrease the stress response^10^. Previous reports demonstrated that abnormal GABA levels in the brain were associated with the pathophysiology of stress syndrome. Indeed, animal trials have shown that lower levels of GABA in the brain are linked to anxiety^11–13^ whilst anxiety is relieved by increasing levels of GABA^13^. Furthermore, these studies have highlighted that the mechanism of action of GABA is related to its ability to lower heat production, reduce sedation, and relieve fever^11–13^. We have shown that in ovo feeding (IOF) of GABA could improve antioxidant status, heat shock protein gene expressions, and lipid metabolism in young and marketable broilers exposed to cyclic HS^14–16^. Another novel approach to HS is embryonic thermal manipulation. This technique consists of exposing the embryo to intermittent high incubational temperatures to confer HS resistance in hatchlings^17^. Previous reports have shown enhanced antioxidant ability, reduced RTs, and lower mortality in ETM birds during thermal challenges^18–20^.

Recently, it was proposed that two or more HS alleviation strategies can be combined to improve the overall effectiveness^21,22^. To our best knowledge, no previous research has been conducted to this date to evaluate the influence of both IOF and ETM on the survival of broilers exposed to HS. The first objective of the current study was to evaluate how ETM and IOF of GABA could affect the core temperature profile of broilers reared under normal and HS conditions (32 ± 1 °C, 4 h daily from days 29 to 35). The second one was to assess how these embryonic treatments could impact the survival of birds when exposed to the same HS challenge.

## Results

The summary of the variables included in the Cox proportional hazard model can be found in Supplementary Table 1.

### Core body temperature dynamics

**Figure 1:**
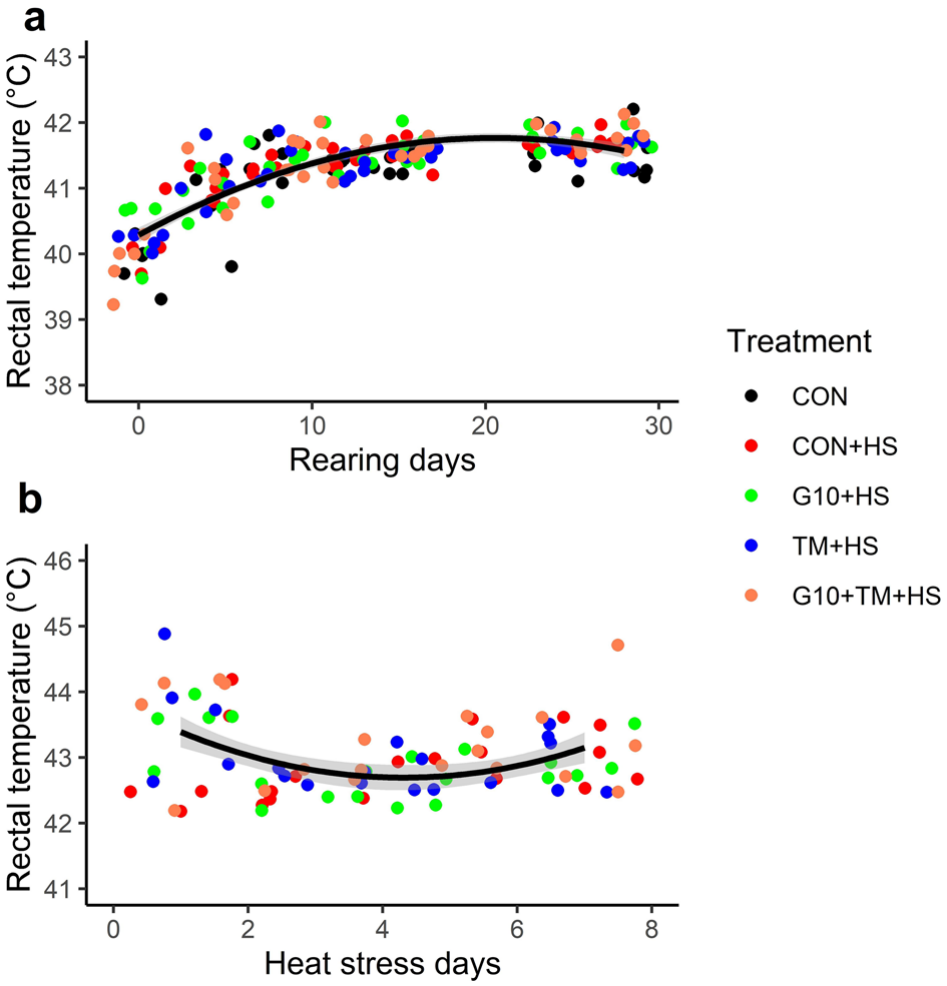
Regression plots of the association between age (Days) and rectal temperatures (RTs) of broilers raised under thermoneutral conditions (**A**) and heat stress (**B**). Polynomial regression analysis revealed a significant quadratic trend for both thermoneutral (y =  − 0.004x^2^ + 0.16x + 40.2, R^2^ = 0.65, P < 0.001) and heat stress (y = 0.063x^2^ − 0.54x + 43.9, R^2^ = 0.41, P = 0.036) conditions. The treatments are as follows: *CON* chicks hatched from control eggs without in ovo injection and incubated at standard temperature, *CON* + *HS* chicks hatched from control eggs without in ovo injection, incubated at standard temperature but exposed to HS, *G10* + *HS* chicks hatched from eggs injected at embryonic day (ED) 17.5 with 0.6 mL of 10% GABA dissolved in distilled water and exposed to HS, *TM* + *HS* chicks hatched from thermally manipulated eggs exposed to 39.6 °C for 6 h daily from ED 10 to 18 and exposed to HS, *G10* + *TM* + *HS* chicks hatched from eggs that received both previous treatments during incubation and exposed to HS.

Table 1 shows the effects of the treatments, day, and their interactions on RT variations in broilers reared under thermoneutral conditions. Neither ETM nor IOF of GABA was shown to influence RTs, but the day had a significant effect (F (6,18) = 67.8, P < 0.001). RTs were the lowest on the day of hatch (day 0) and gradually increased to a plateau on day 8, after which there were no significant differences between the days. Consistent with these results, polynomial regression revealed a quadratic effect (R^2^ = 0.65, P < 0.001) of day on RT changes (Fig. 1a). HS challenge influenced RTs depending on both day and treatment (Table 2). Compared with CON, the HS challenge significantly increased RT by 1.6–2.3 °C, regardless of GABA, TM, or their combination (F (4,20) = 45.4, P < 0.001). Besides, orthogonal contrast also revealed that on day 3 of HS, the birds hatched after ETM (TM + HS and G10 + TM + HS) had significantly higher RTs compared with the CON + HS group (F (1,13) = 7.01, P = 0.015). During the first day of HS, the birds registered higher (F (3,21) = 7.52, P < 0.001) RTs compared to 3 and 5 days of HS, but no differences were found when compared to day 7 of HS. Thus, there was a quadratic effect (R^2^ = 0.41, P < 0.05) of day on RT by polynomial regression (Fig. 1b).

**Table 1:** Effects of embryonic thermal manipulation (ETM) and in ovo feeding (IOF) of r-aminobutyric acid (GABA) (G10) on rectal temperature (RT) of broilers reared under thermoneutral conditions.

Rearing days	CON	Treatments					Total
		CON	CON	–	–	–	
	G10	–	–	G10	–	G10	
	TM	–	–	–	TM	+ TM	
	HS	–	+ HS	+ HS	+ HS	+ HS	
Day 0		39.9 ± 0.2	39.7 ± 0.6	40.3 ± 0.2	40.2 ± 0.1	39.8 ± 0.2	39.9 ± 0.2^A^
Day 4		41.4 ± 0.0	41.4 ± 0.1	41.5 ± 0.1	41.3 ± 0.1	41.6 ± 0.2	41.0 ± 0.1^AB^
Day 8		41.4 ± 0.1	41.6 ± 0.1	41.6 ± 0.1	41.6 ± 0.0	41.6 ± 0.1	41.4 ± 0.1^B^
Day 12		41.5 ± 0.2	41.6 ± 0.0	41.8 ± 0.1	41.6 ± 0.1	41.7 ± 0.1	41.4 ± 0.1^B^
Day 16		41.5 ± 0.2	41.7 ± 0.1	41.7 ± 0.1	41.6 ± 0.1	41.9 ± 0.1	41.6 ± 0.1^B^
Day 24		40.8 ± 0.3	41.0 ± 0.1	40.9 ± 0.1	41.2 ± 0.2	41.1 ± 0.2	41.6 ± 0.1^B^
Day 28		41.5 ± 0.1	41.4 ± 0.1	41.3 ± 0.2	41.5 ± 0.2	41.4 ± 0.1	41.7 ± 0.1^B^
Total		41.1 ± 0.1	41.2 ± 0.1	41.3 ± 0.2	41.3 ± 0.2	41.3 ± 0.1	NA
P-value							
Treatment (T)		0.211					
Day (D)		< 0.001					
T × D		0.837					

The treatments are described as follows: *CON* chicks hatched from control eggs without in ovo injection and incubated at standard temperature, *CON* + *HS* chicks hatched from control eggs without in ovo injection, incubated at standard temperature but exposed to HS, *G10* + *HS* chicks hatched from eggs injected at 17.5 days of incubation with 0.6 mL of 10% GABA dissolved in distilled water and exposed to HS, *TM* + *HS* chicks hatched from thermally manipulated eggs exposed to 39.6 °C for 6 h daily from ED 10 to 18 and exposed to HS, *G10* + *TM* + *HS* chicks hatched from eggs that received both previous treatments during incubation and exposed to HS. Data show mean ± SEM (n = 5).

Means bearing different superscripts indicate significant differences by day (A, B) effect (P < 0.05).

**Table 2:** Effects of embryonic thermal manipulation (ETM) and in ovo feeding (IOF) of r-aminobutyric acid (GABA) (G10) on rectal temperature (RT) of broilers exposed to heat stress (HS).

Days of HS	CON	Treatments					Total
		CON	CON	–	–	–	
	G10	–	–	G10	–	G10	
	TM	–	–	–	TM	+ TM	
	HS	–	+ HS	+ HS	+ HS	+ HS	
Day 1		41.4 ± 0.2	43.0 ± 0.4	43.5 ± 0.2	43.6 ± 0.4	43.7 ± 0.4	43.0 ± 0.2^A^
Day 3		41.5 ± 0.1	42.5 ± 0.1	42.5 ± 0.1	42.7 ± 0.0	42.8 ± 0.1	42.4 ± 0.1^B^
Day 5		41.4 ± 0.2	43.1 ± 0.2	42.7 ± 0.2	42.8 ± 0.1	43.2 ± 0.2	42.6 ± 0.1^B^
Day 7		41.3 ± 0.1	43.1 ± 0.2	42.9 ± 0.1	43.0 ± 0.2	43.3 ± 0.4	42.7 ± 0.2^AB^
Total		41.4 ± 0.1^a^	42.9 ± 0.1^b^	42.9 ± 0.1^b^	43.0 ± 0.1^b^	43.3 ± 0.2^b^	NA
P-value							
Treatment (T)		< 0.001					
Day (D)		< 0.001					
T × D		0.281					
Contrast		CON + HS vs G10 + HS and G10 + TM + HS			CON + HS vs TM + HS and G10 + TM + HS		
Day 1		0.143			0.121		
Day 3		0.109			0.015		
Day 5		0.445			0.609		
Day 7		0.862			0.755		

The treatments are described as follows: *CON* chicks hatched from control eggs without in ovo injection and incubated at standard temperature, *CON* + *HS* chicks hatched from control eggs without in ovo injection, incubated at standard temperature but exposed to HS, *G10* + *HS* chicks hatched from eggs injected at 17.5 days of incubation with 0.6 mL of 10% GABA dissolved in distilled water and exposed to HS, *TM* + *HS* chicks hatched from thermally manipulated eggs exposed to 39.6 °C for 6 h daily from embryonic day (ED) 10 to 18 and exposed to HS, *G10* + *TM* + *HS* chicks hatched from eggs that received both previous treatments during incubation and exposed to HS. Data show mean ± SEM (n = 5). Means bearing different superscripts indicate significant differences by day (A.B) and treatment (a,b) effect (P < 0.05).

**Figure 2:**
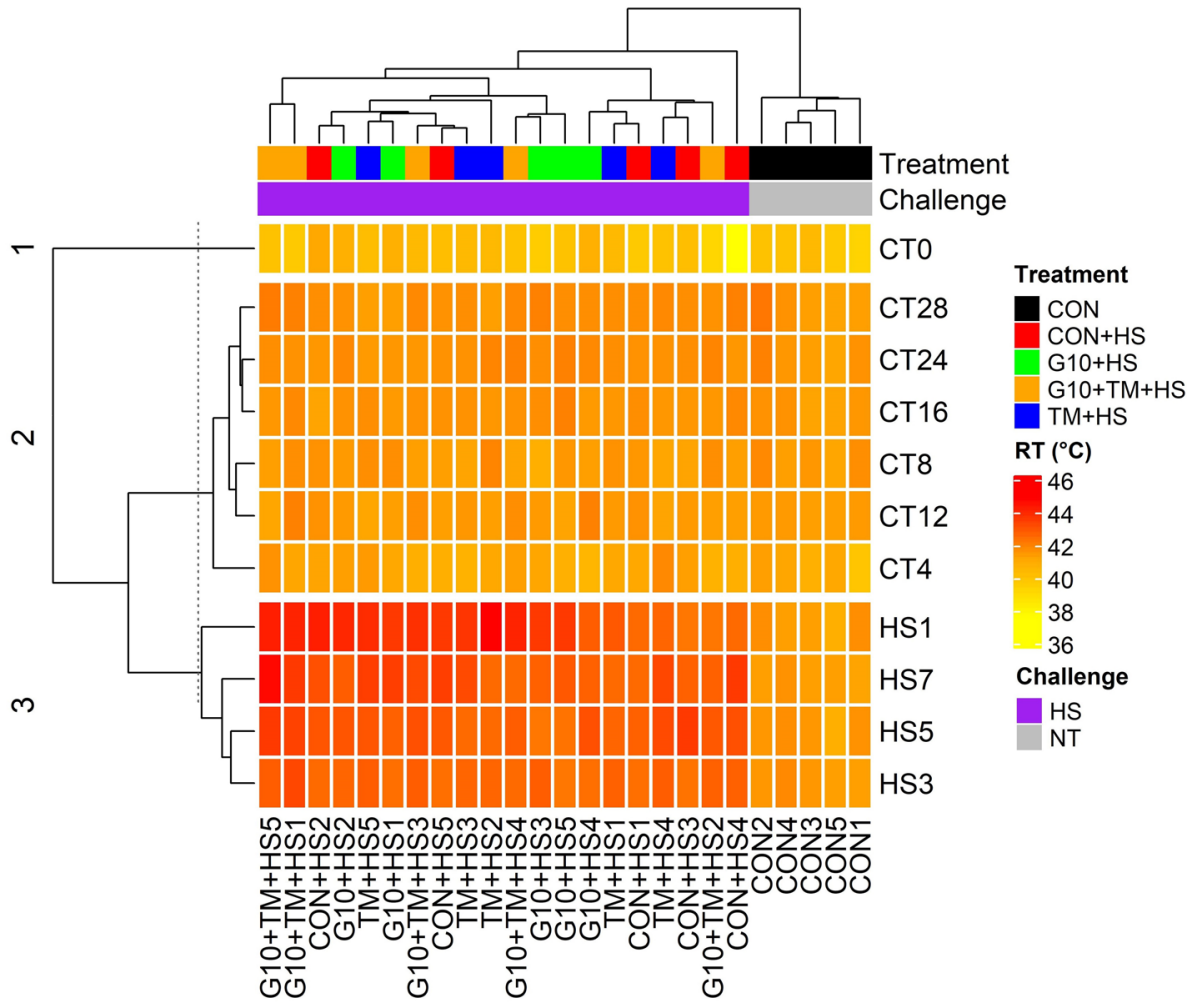
A clustered heatmap for rectal temperatures in broilers exposed to high ambient temperatures. Temperature panels included RTs at thermoneutral (NT) and heat stress conditions (HS). The heatmap was constructed using the package “ComplexHeatmap” of the R software version 4.0.3 (R Core Team, 2020). Each row represents an RT record and each column represents an experimental unit belonging to a specific treatment. Precisely, CTX refers to RT records at a day X of thermoneutral condition; similarly, HSX refers to RT records at a day X of HS challenge. The treatments are as follows: *CON* chicks hatched from control eggs without in ovo injection and incubated at standard temperature, *CON* + *HS* chicks hatched from control eggs without in ovo injection, incubated at standard temperature but exposed to HS, *G10* + *HS* chicks hatched from eggs injected at embryonic day (ED) 17.5 with 0.6 mL of 10% GABA dissolved in distilled water and exposed to HS, *TM* + *HS* chicks hatched from thermally manipulated eggs exposed to 39.6 °C for 6 h daily from ED 10 to 18 and exposed to HS, *G10* + *TM* + *HS* chicks hatched from eggs that received both previous treatments during incubation and exposed to HS.

The RT data were clustered into three groups based on Euclidean distances (Fig. 2). The first cluster was for the RTs of day-old birds, the lowest among other ages. The second one included all RTs during the standard rearing conditions except those for the first cluster. The last was for the RTs measured during the HS challenge, where the RTs were highest among the clusters.

**Figure 3:**
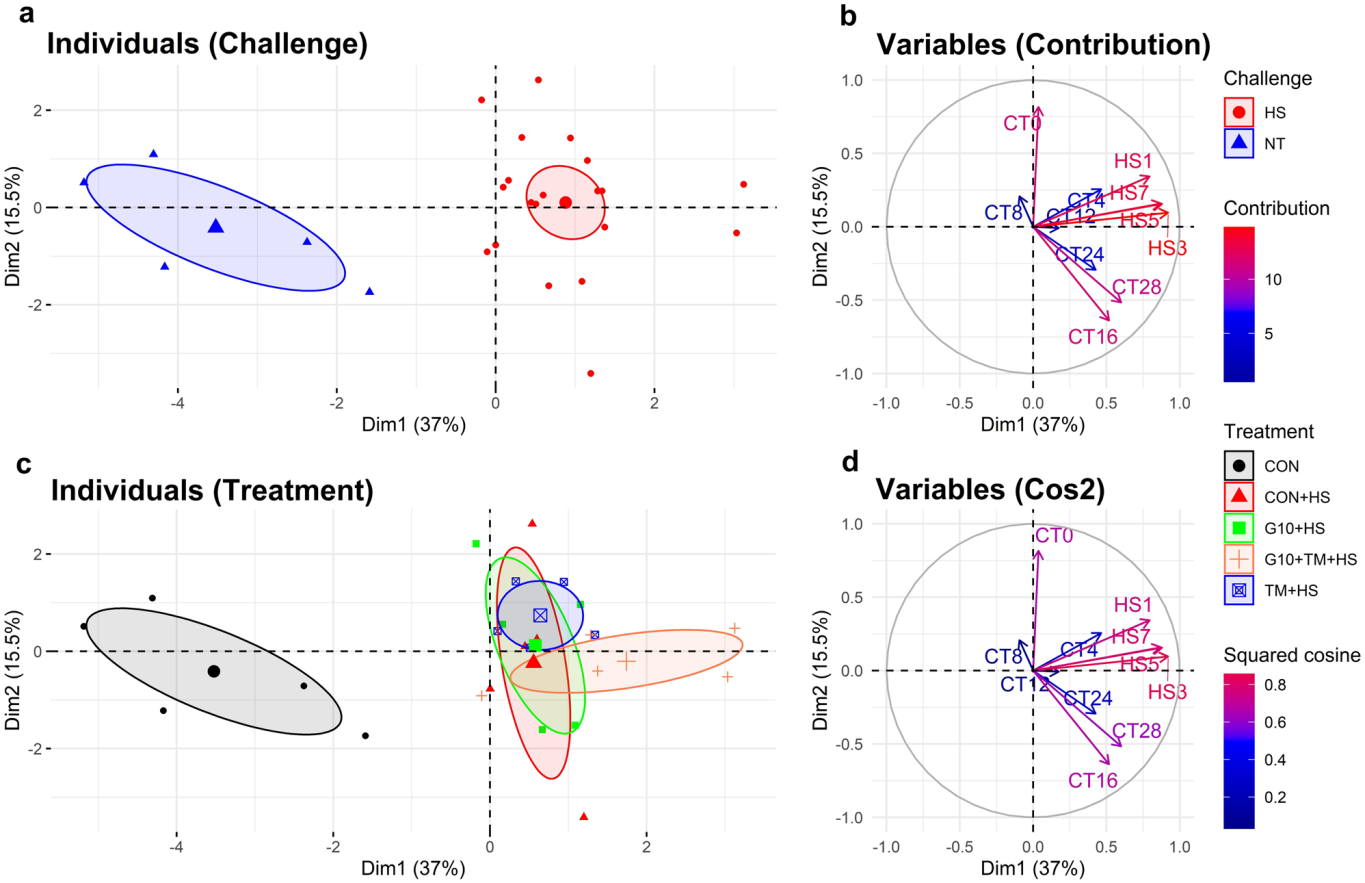
Principal component analysis (PCA) plot of individuals and variables. An individual refers to a replicate per treatment while a variable is a rectal temperature (RT) record. The individuals have been colored according to the challenge (**A**) and treatments (**C**). The variables have been colored based on their contribution (**B**) or their squared cosine (**D**). Precisely, CTX refers to RT records at a day X of thermoneutral condition; similarly, HSX refers to RT records at a day X of HS challenge. The PCA was executed in R using the package “FactoMineR”. Multivariate analysis of variance (MANOVA) based on the first 3 dimensions of the PCA explaining 65.9% of variability revealed that treatment and challenge had a significant influence on data clustering. The challenges are described as follows: *NT* thermoneutral temperature, *HS* heat stress. The treatments are as follows: *CON* chicks hatched from control eggs without in ovo injection and incubated at standard temperature, *CON* + *HS* chicks hatched from control eggs without in ovo injection, incubated at standard temperature but exposed to HS, *G10* + *HS* chicks hatched from eggs injected at embryonic day (ED) 17.5 with 0.6 mL of 10% GABA dissolved in distilled water and exposed to HS, *TM* + *HS* chicks hatched from thermally manipulated eggs exposed to 39.6 °C for 6 h daily from ED 10 to 18 and exposed to HS, *G10* + *TM* + *HS* chicks hatched from eggs that received both previous treatments during incubation and exposed to HS. The dotted line indicates the level at which the K-means algorithm truncates the dendrogram (in this case, the line cuts to show how the three groups are separated).

The three first components (Dim 1, Dim 2, and Dim 3) of the PCA analysis accounted for 37%, 15.5%, and 13.4%, respectively, of the total variability contained in the dataset. Besides, the MANOVA procedure conducted on these three components revealed a significant effect of both treatments (F (12,60) = 2.39, P = 0.013) and thermal challenge (F (3,21) = 26.1, P < 0.001) (Fig. 3a,c). The PCA showed correspondence between the RTs recorded during the HS challenge, which contributed the most to the first principal component (Fig. 3b,d). On the other side, the RTs recorded on hatch day (CT0), day 16 (CT16), and day 28 (CT28) were the variables that contributed the most to the construction of the second component (Fig. 3b,d). Thus, individuals represented on the left side of the plan (Fig. 3a) were the birds that recorded the lowest RTs during the HS challenge. As expected, they all belonged to the CON group. By contrast, the birds from the G10 + TM + HS group were located on the extreme right of the plan, suggesting that they had the overall highest RTs during the HS challenge (Fig. 3c).

**Figure 4:**
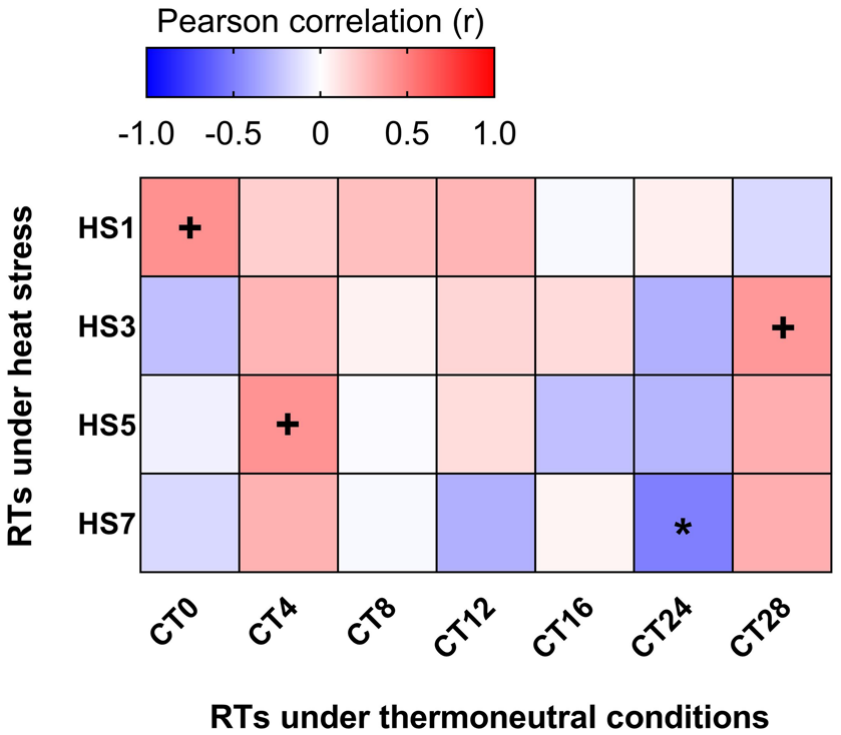
Pearson correlation heat map between the RTs under thermoneutral conditions (CT), and RTs under heat stress (HS) in broiler chickens. Each row represents an RT record during heat stress and each column represents an RT record during thermoneutral temperature. Precisely, CTX refers to RT records at a day X of thermoneutral condition; similarly, HSX refers to RT records at a day X of HS challenge. The red color indicates a positive correlation, the blue color indicates a negative correlation and the white color indicates no correlation. Pearson r values were calculated using the “CORR procedure” of the SAS software version 9.4 (SAS Institute Inc., 2009). ^+^Indicates a trend at P < 0.1 level. *Correlation is significant at P < 0.05 level.

Finally, Fig. 4 indicates that Pearson correlation analysis highlighted a negative significant correlation (r =  − 0.50, P = 0.032) between the RT on day 24 (CT24) and the RT during the last day of HS (HS7).

### Hazard of death in heat-stressed birds

**Figure 5:**
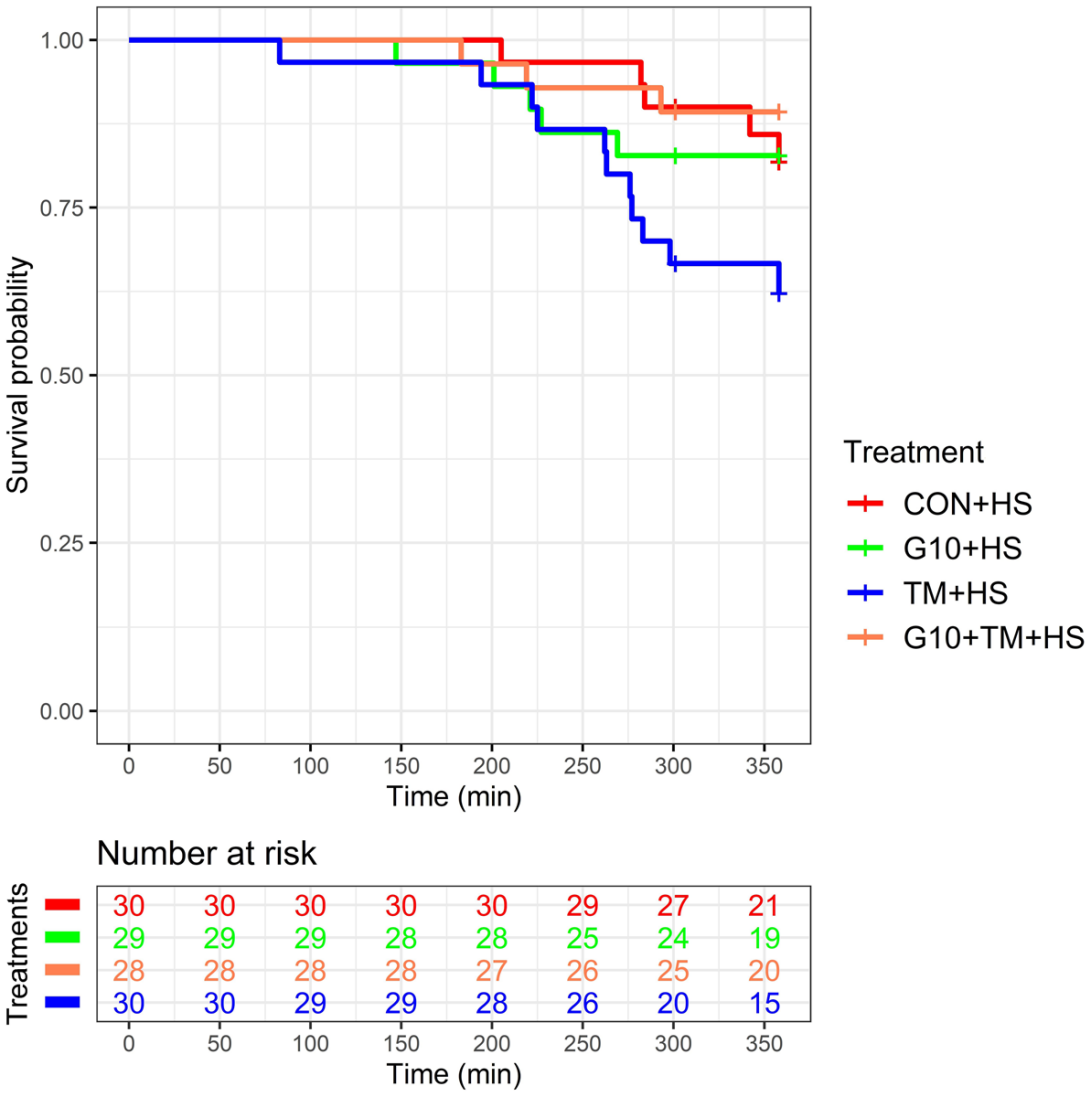
Kaplan-Meir survival curves and risk table of broilers exposed to HS. At t = 301 min, 5 birds per treatment were censored for biological sampling. The x-axis (Time) represents the time elapsed during the HS challenge. The “Number at risk” refers to the number of birds alive at a specific time per treatment. The treatments are as follows: *CON* chicks hatched from control eggs without in ovo injection and incubated at standard temperature, *CON* + *HS* chicks hatched from control eggs without in ovo injection, incubated at standard temperature but exposed to HS, *G10* + *HS* chicks hatched from eggs injected at embryonic day (ED) 17.5 with 0.6 mL of 10% GABA dissolved in distilled water and exposed to HS, *TM* + *HS* chicks hatched from thermally manipulated eggs exposed to 39.6 °C for 6 h daily from ED 10 to 18 and exposed to HS, *G10* + *TM* + *HS* chicks hatched from eggs that received both previous treatments during incubation and exposed to HS.

Kaplan-Meir survival curve and the risk table indicate the survival probability of chicks over time (Fig. 5). The CON + HS, G10 + HS, TM + HS, and G10 + TM + HS had 30, 29, 30, and 28 chicks, respectively, at the beginning of the HS challenge. At t = 301 min, 5 birds per treatment were censored for biological sampling. Thus, mortality after HS was estimated at 16.7%, 17.2%, 36.7%, and 10.7% in the corresponding treatments.

**Figure 6:**
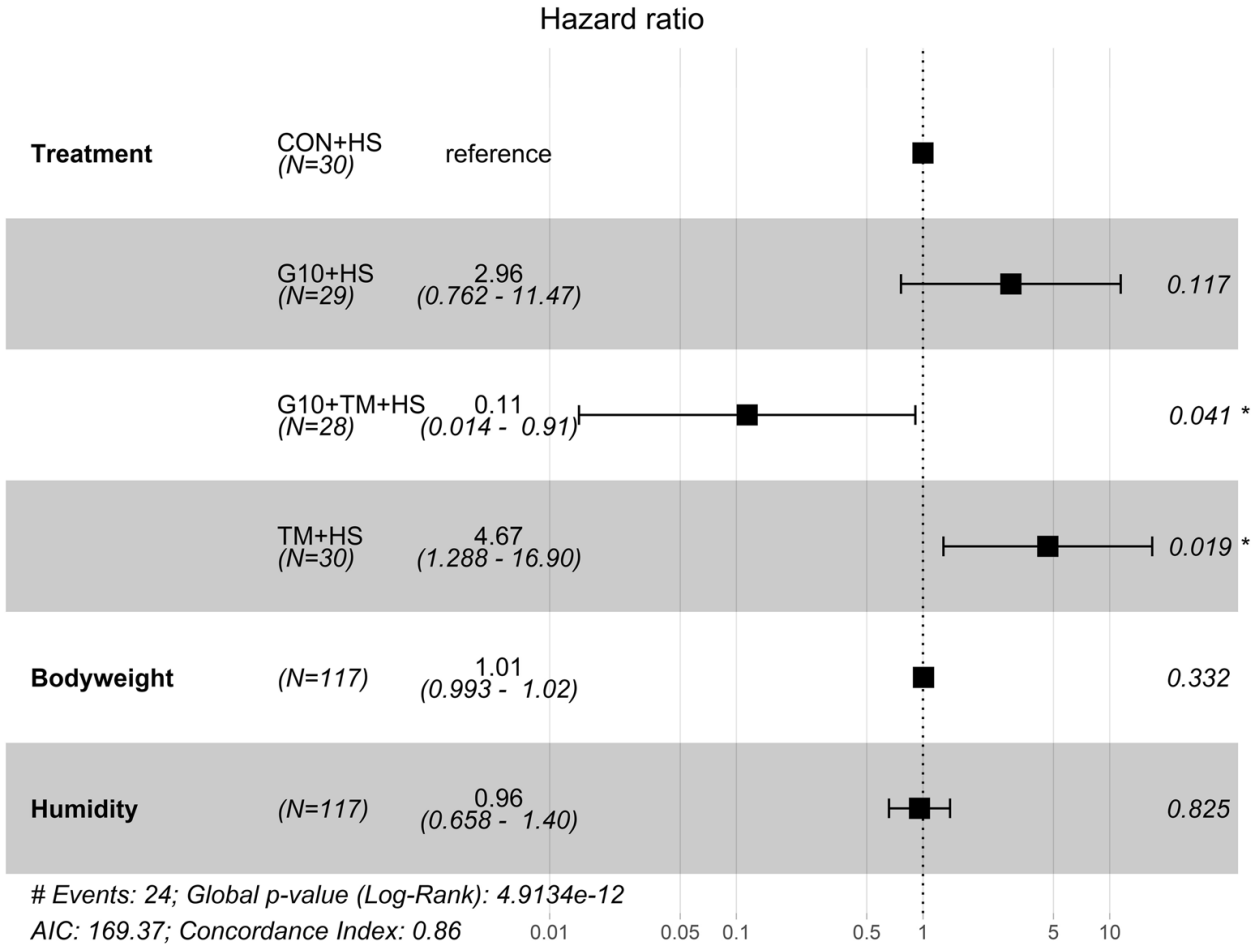
Forest plot of the Cox proportional-hazards model of broilers chickens exposed to cyclic heat stress. The model was built with the package “Survminer” of the R software version 4.0.3 (R Core Team, 2020). Covariates retained in the model are presented on the left with bolded letters. The vertical dashed line refers to a hazard ratio of 1. The black squares and their intervals refer respectively to the hazard ratios and 95% confidence intervals associated with each covariate. A hazard ratio above 1 indicates a positive association with death; a hazard ratio below 1 indicates a reduction in the probability of death. The treatments are as follows: *CON* chicks hatched from control eggs without in ovo injection and incubated at standard temperature, *CON* + *HS* chicks hatched from control eggs without in ovo injection, incubated at standard temperature but exposed to HS, *G10* + *HS* chicks hatched from eggs injected at embryonic day (ED) 17.5 with 0.6 mL of 10% GABA dissolved in distilled water and exposed to HS, *TM* + *HS* chicks hatched from thermally manipulated eggs exposed to 39.6 °C for 6 h daily from ED 10 to 18 and exposed to HS, *G10* + *TM* + *HS* chicks hatched from eggs that received both previous treatments during incubation and exposed to HS.

Figure 6 presents the forest plot summarizing the covariates that were retained after the stepwise selection of variables in the Cox proportional hazard model. The forest plot is used to graphically represents the level of significance of the estimated effect of a covariate on birds’ survival. Stepwise selection is a technique that uses an automatic procedure to select the covariates among all (treatment, humidity, temperature, THI, bodyweight) ensuring a better fit of the Cox model. The covariates selected were treatment, bodyweight, and humidity. The estimated hazard ratio (HR) and its confidence interval (CI) can be used as indicators of the effect of each covariate. Compared with the CON + HS group, the TM + HS group were significantly more likely (HR 4.67, 95% CI 1.28 to 16.9, P = 0.019) to die, but the G10 + TM + HS group had a significantly lower risk of death (HR 0.11, 95% CI 0.01 to 0.91, P = 0.041). Even though they were included in the model, no significant effects of the humidity (HR 0.96, 95% CI 0.66 to 1.40, P = 0.825) and bodyweight (HR 1.01, 95% CI 0.99 to 1.02, P = 0.332) were detected.

## Discussion

Global climate change has emerged as an important agenda in international politics and the livestock industry. Meat-type chickens are more likely to be susceptible to high ambient temperatures due to the lack of sweat glands, the presence of feathers, and their fast growth rate^23^. In this study, we investigated the effects of thermoneutral and HS conditions on RT change in broilers and ETM and GABA IOF on their survival.

Under standard rearing conditions, RTs gradually increased, with a minimum at hatching days followed by consistently higher levels from days 8 to 28 (Fig. 1a). In this study, neither IOF of GABA nor ETM affected RTs during the first 28 days. Our results are consistent with a previous study that did not detect any differences in RT between control and thermally manipulated Hubbard chicks after a 42-day trial^24^. However, others have shown the opposite results. For example, ETM significantly reduced RTs of Hubbard and Cobb broilers raised under thermoneutral temperature^25^. Similarly, lower RTs were reported in Ross chicks from days 7 to 28 when ETM was applied for 3 days between EDs 16 and 18^26^. When evaluating the effects of ETM on changes in body temperatures in birds, it is also desirable to consider parameters such as breed, temperature, and duration of manipulation. Overall, the discrepancy in the literature on the effect of ETM on broiler RTs is too large to draw conclusions.

Under HS, chickens act to maintain body temperature by drinking water, panting, or spreading their wings^27^. As seen in the current study, increases in the ambient temperature can critically influence the body temperature of chickens. RTs exhibited a similar rise with increasing ambient temperature (Fig. 1b). PCA revealed a clear separation between the birds in thermoneutral and HS environments (Fig. 3a). This separation was mainly located in the first dimension of PCA, which was highly correlated with the RTs of HS (Fig. 3b,d). These results are consistent with previous reports that mentioned augmented heat loads concomitant with disturbed thermal balance during HS^28,29^. Interestingly, the RTs on days 3 and 5 of the HS challenge were significantly lower than those for day 1. The increase in RTs in the initial phase of exposure may be due to the shock state which becomes more stable over time (54–66 h) and then decreases^29^. Chickens had lower RTs after 24 h of continuous heat exposure^29^ or after 2 weeks of HS exposure following the initiation of a chronic HS^30^. These results suggest that behavioral changes such as panting, spreading wings, and increasing water intake in birds during heat exposure may help to lower internal temperatures to a limited extent^31^. Enhanced water intake may especially alleviate birds with increased respiration rates due to HS^27,29^. It is also important to mention that GABA IOF and ETM did not influence RTs during HS as previously observed in thermoneutral conditions.

We used survival analysis to firstly identify the likelihood of death of birds belonging to a specific treatment, and secondly to point out factors associated with mortality under high ambient temperature. Survival analysis is a tool to detect critical periods or factors associated with death by comparing risk patterns under different conditions^32^. Since mortality only occurred within a few hours of the first day of exposure to HS in our experimental setting, it is important to clarify that in the current trial survival times were expressed in minutes. Because the temperature rise occurred within 30 min, it is likely that the birds were not able to adopt behavioral changes quickly enough to cope with HS. This might have caused the weakest birds to die during the early phase of HS. As feed intake affects metabolism and thus body temperature^33^, elevated core body temperatures were linked to higher feed intake and increased thermogenesis per gram of feed^34^. Another reason why mortality did not occur after the first day of HS may be accounted for by acclimatization. During periodic HS, hot periods such as summer alternate with normal rearing temperatures within a day, increasing the broiler’s coping ability over time^35^.

Even though the RTs during HS did not significantly differ across treatments, PCA results suggest that the G10 + TM + HS group had the highest RTs (Fig. 3c) but was the least likely to die due to HS (Fig. 6). In other words, the G10 + TM + HS group was able to tolerate overall higher core body temperature with minimal lethal effect. Therefore, the improved HS resistance observed after combining both IOF of GABA and ETM was not associated with decreased metabolic rate as previously described^24,25^. In addition, the higher survival of G10 + TM + HS treatment should mostly be due to the IOF of GABA rather than ETM, as the TM + HS group had the least resilience to HS. During HS the increase in body temperature accelerates the metabolism of the birds^36^. To preserve homeostasis, there is an increase in energy requirements which is manifested by the active stimulation of glucose metabolism and the suppression of lipid metabolism^37,38^. Our previous studies have already shown that the IOF of GABA provides thermotolerance in young and market-age broilers by regulating lipid metabolism and enhancing antioxidant functions during HS^14,15^. On the other side, the TM + HS group had a lower probability of survival. ETM is postulated to confer HS resistance because embryos are pre-exposed to similar heat waves that chicks may encounter later in life, which may activate thermoregulatory memories^39,40^. However, since in the current trial, the HS regimen and the ETM protocol were not similar, it may have contributed to the observed results. Although overall mortality was within the same range between the CON + HS, G10 + HS, and G10 + TM + HS groups, the probability of death during HS challenge differed mainly due to the time within each death record (Fig. 5). For instance, the first death occurred relatively late in both the G10 + TM + HS and CON + HS groups, but the number of dead chickens became increasingly sparse over time in the G10 + TM + HS group. Survival analysis takes into consideration both overall mortality and death rate (which is a function of time). This approach gives much more power to detect differences between groups^41^. Thus, populations with similar mortalities might have different survival probabilities. To our best knowledge, the use of survival analysis to evaluate HS survival of broilers after ETM or GABA IOF has not yet been done before. A positive effect of ETM on antioxidant status or heat shock protein gene expression in heat-stressed broilers has already been shown^19,20,42^, but its effect on their survival remains unclear. The disparity between the results of this report and previous studies resides in the methodology used to assess the effectiveness of ETM treatment. In fact, several authors have evaluated the differences in mortality between control and ETM birds using a chi-square test and found no or slightly lower mortality after ETM^20,43,44^. However, our study included time and other covariates in the equation. Overall, the improved survival in the G10 + TM + HS suggests that the combination of both ETM and GABA IOF may be more effective than taking each treatment individually.

Since several variables were recorded during the trial, the stepwise method was used to semi-automatically select the variable included in the model. This method has been previously used in Cox regression for its versatility and strength in detecting associations amongst covariates and cofounding^45^. Thus, the model including treatment, humidity, and bodyweight was selected due to better explanatory power (based on AIC and c-index).

In conclusion, From the results of the current trial, it has been revealed that in broilers reared under thermoneutral temperature, body core temperature tends to increase quadratically with age. In addition, the early stages of HS are the most critical moments for birds since their RTs tend to slightly decrease with a longer period of thermal exposure. It also appears that the combination of GABA IOF and ETM improved broilers’ survival at high rearing temperatures. However, further studies are needed to understand the biological mechanism behind the combined action of GABA IOF and ETM.

## Materials and methods

### Ethics statement

The experimental procedures for this study were approved by the Institutional Animal Care and Use Committee of Gyeongsang National University (GNU-200916-C0058) and were in compliance to the latest version of the ARRIVE guidelines^46^. All the methods were performed in accordance with the relevant guideline and regulations.

### Incubation, in ovo feeding and thermal manipulation procedures

Further details concerning the incubation conditions, the IOF procedure, as well as the ETM, can be found in previously published articles^14,47^. Briefly, eggs (n = 300) were obtained from 37-week-old Indian River breeder hens in a local broiler breeder farm (Hapcheon, Korea) and were set in two identical incubators (Maru 190, Rcom Co., Ltd., Gimhae, Korea). After candling at embryonic day (ED) 10, non-fertilized eggs were removed from the incubators, and the eggs were distributed into one of five groups (n = 48 eggs per group) of similar weight using the Solver module of Microsoft Excel (Microsoft Excel 2016; Microsoft Corp., Redmond, WA, USA). One incubator used a standard incubational condition which was 37.8 °C and 56% of relative humidity (RH) from EDs 1 to 17 and 36.8 °C and 70% RH from EDs 18 to 21. The incubator had three groups of eggs. Eggs from two groups were used as controls without any treatment, while the remaining group received an IOF of 0.6 mL of 10% GABA (#A2129, Sigma-Aldrich, Inc., St. Louis, MO, USA) dissolved in distilled water at ED 17.5. IOF procedure was as follows. Eggs were disinfected with a 70% ethanol solution, and a small hole was drilled on the surface of the large end of the egg using a dental drill (Saeshin, Daegu, Korea). Thereafter, an injection, targeting the amniotic sac, was made via the hole using a 1 mL syringe with a 23G, 25 mm needle. The other incubator contained the two remaining groups that were used to perform ETM. ETM was performed by increasing the temperature from 37.8  to 39.6 °C for 6 h daily from EDs 10 to 18. One group of eggs was only subjected to ETM while the other also received both ETM and IOF of GABA.

### Feeding trial and HS challenge

**Figure 7:**
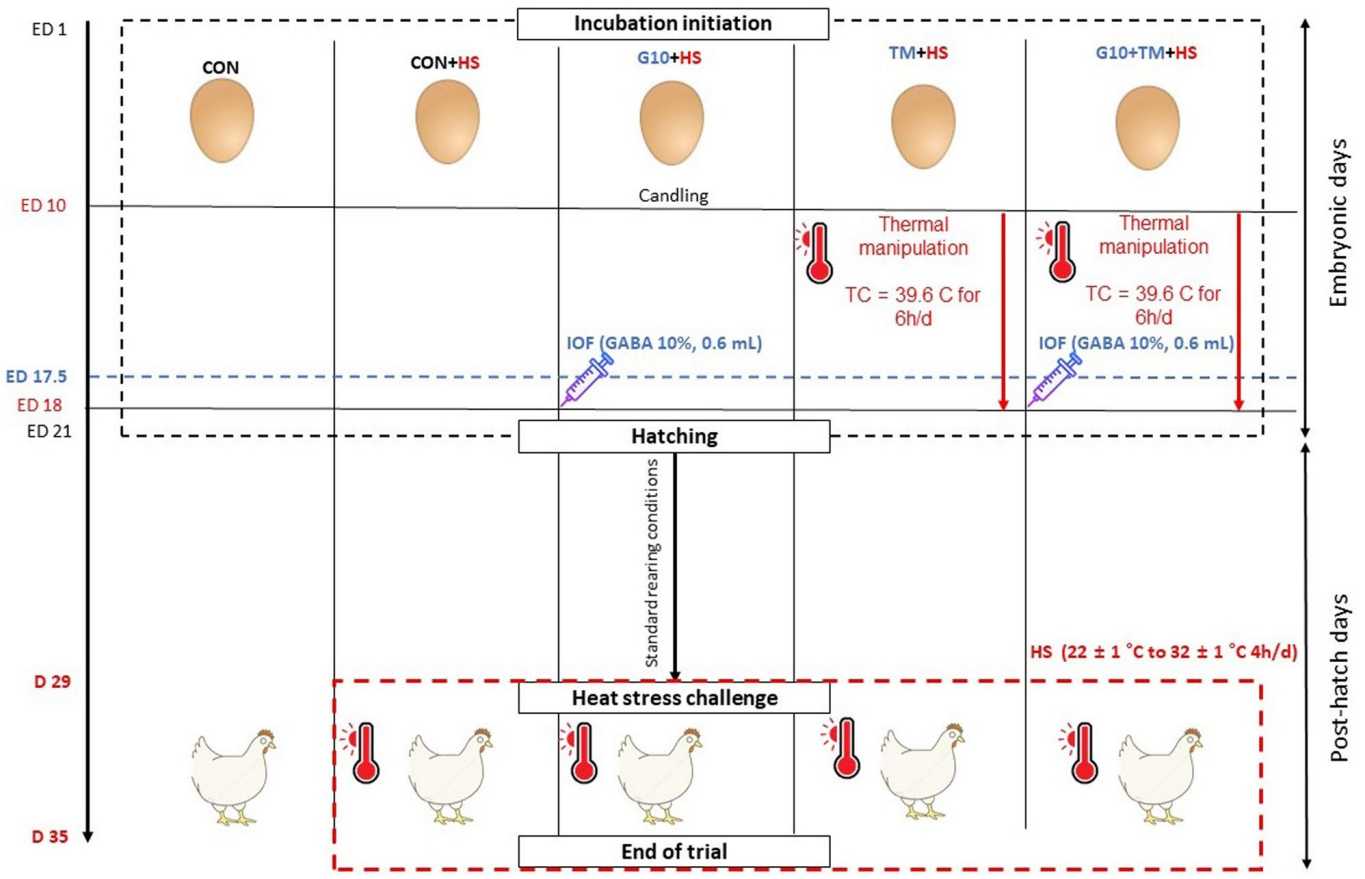
Study design. The treatments are described as follows: *CON* chicks hatched from control eggs without in ovo injection and incubated at standard temperature, *CON* + *HS* chicks hatched from control eggs without in ovo injection, incubated at standard temperature but exposed to HS, *G10* + *HS* chicks hatched from eggs injected at 17.5 days of incubation with 0.6 mL of 10% GABA dissolved in distilled water and exposed to HS, *TM* + *HS* chicks hatched from thermally manipulated eggs exposed to 39.6 °C for 6 h daily from ED 10 to 18 and exposed to HS, *G10* + *TM* + *HS* chicks hatched from eggs that received both previous treatments during incubation and exposed to HS.

After hatching, a total of one hundred fifty unsexed Indian River day-old chicks were raised in battery brooders under a thermally controlled environment at 34 ± 1 °C and 50% RH, and then the temperature was gradually decreased by 2 or 1 °C every 3 days to reached the recommended 22 ± 1 °C on day 28^48^. A graphical representation can be found for the temperature change during the feeding trial in Supplementary Fig. 1. A commercially available feed and water were provided ad libitum under 23 h of light (from 01:00 h to 24: 00 h) and 1 h of dark daily. On day 29, the chicks were allocated into five different treatment (5 replicates of 6 birds) groups: chicks hatched from control eggs without IOF and incubated at standard temperature (CON); chicks hatched from control eggs without IOF, incubated at standard temperature but exposed to HS (CON + HS); chicks hatched from eggs that received IOF of GABA and exposed to HS (G10 + HS); chicks hatched from thermally manipulated eggs and exposed to HS (TM + HS); chicks hatched from eggs that received both IOF of GABA and ETM then exposed to HS (G10 + TM + HS). The birds were challenged with a cyclic HS between 29 and 35 days of age following a previously executed protocol with minor modification^49^. An overview of the design is presented in Fig. 7. The room temperature modification was performed every day during the same period (from 14:00 h to 19:00 h) to ensure consistency in the design. In the HS room, the environmental temperature was gradually increased from 22 ± 1 to 32 ± 1 °C over 30 min, and this temperature was maintained for the next 4 h before returning to 22 ± 1 °C over 30 min. Meanwhile, chicks reared at thermoneutral temperature (CON) were kept at 22 ± 1 °C until day 35 (Supplementary Table 2).

### RT and bodyweight measurement

One bird from each cage was randomly selected for RT measurement. RT was recorded using a digital thermometer (HANNA instruments Inc., Padova, Italy) with the probe inserted into the cloaca until the temperature readings stabilized. During standard rearing conditions, RT was recorded at the same time of the day (17:00 h) on the hatch, days 4, 8, 12, 16, 24, and 28 to minimize potential sampling errors. Similarly, RT was recorded at 17:00 h every 2 days from the beginning (day 29) to the end (day 35) of the HS challenge, but individual birds were weighed just before and after the challenge.

### Estimation of the survival

HS survival time (measured in min), the main outcome variable of the current trial was estimated from the beginning of the HS challenge (day 29) to the time of death or censoring. In survival analysis, censoring is common and refers to a form of missing data in which time to event is not observed for a specific reason. In this trial, at t = 301 min the censored birds were euthanized for biological sample collection. Thus, the survival time was not collected for 5 birds per treatment. Variables such as temperature, humidity, and bodyweight of the birds were recorded whenever a bird died and at the end of the trial (t = 358 min). In addition, we also calculated the temperature-humidity index (THI) according to the following formula^50^:

1 $${\text{THI}}_{{{\text{broilers}}}} = \, 0.{\text{85 T}}_{{\text{dry bulb}}} + \, 0.{\text{15 T}}_{{\text{wet bulb}}} .$$

### Statistical analysis

Before analysis, Shapiro Wilk’s and Levene’s tests were conducted to assess the normality and homoscedasticity assumptions. RTs were analyzed via a two-way ANOVA procedure with treatment and day as the main effects and their interactions. When a significant p-value was found, a Tukey post hoc test was conducted to evaluate differences among means. To analyze the RTs during HS, orthogonal contrasts were made to evaluate the specific effects of ETM (CON + HS vs TM + HS and G10 + TM + HS) and GABA IOF (CON + HS vs G10 + HS and G10 + TM + HS). In addition, day-related effects were assessed using polynomial regression analysis. Pearson correlations were also calculated between RTs.

Concerning multivariate analysis, principal component analysis (PCA) was performed with RTs as the active variables, and treatment and heat challenge as supplementary variables. Thereafter, a one-way MANOVA was applied on the three first dimensions of the PCA to detect differences between the centroids of the cluster of samples belonging to each treatment^51^. Furthermore, a hierarchical cluster tree was constructed based on Euclidian distances to detect similarities in the RTs dataset.

Finally, a forward selection stepwise Cox regression was conducted to determine the optimal model for evaluating the effects of treatment, temperature, relative humidity, THI, and broiler’s bodyweight on their survival. Schoenfeld and Martingale residuals were used to determine proportional hazards and the nonlinearity relationship between log hazard and covariates assumptions. The model’s equation was:

2 $$h\left( {\text{t}} \right) \, = h_{0} \left( {\text{t}} \right) \, \times {\text{ exp }}\left( {b_{{1}} x_{{1}} + b_{{2}} x_{{2}} + \cdots + b_{{\text{n}}} x_{{\text{n}}} } \right),$$

where *h*(t) is the hazard function determined by the covariates (*x*_1_, *x*_2_, …, *x*_n_), which are treatments, temperature, humidity, THI, and body weight in the current study; the terms (*b*_1_, *b*_2_, …, *b*_n_) are the regression coefficients that quantify the association between each covariate and the hazard; t is the survival time, and *h*_0_ (t) is the baseline hazard representing the hazard when all the covariates are equal to zero. The model was selected based on the lowest Akaike Information Criterion (AIC) and the highest concordance index (c-index). Two-way ANOVA, polynomial regression analysis, orthogonal contrasts, and Pearson correlation were performed using the SAS software (v.9.4) (SAS Institute Inc., 2009). The “FactoMineR”, “Complexheatmap”, and “Survminer” packages of the R software (v.4.0.3) (R Core Team, 2020) were used to conduct PCA, MANOVA, hierarchical clustering, and survival analysis.

## Supplementary Information

Supplementary Information.

## Data Availability

All datasets obtained and analyzed in this research are included in this article.

## References

[1] Nawab A (2018). Heat stress in poultry production: Mitigation strategies to overcome the future challenges facing the global poultry industry. J. Therm. Biol..

[2] Goel A, Ncho CM, Choi YH (2021). Regulation of gene expression in chickens by heat stress. J. Anim. Sci. Biotechnol..

[3] Donkoh A (1989). Ambient temperature: A factor affecting performance and physiological response of broiler chickens. Int. J. Biometeorol..

[4] Teeter RG, Belay T (1996). Broiler management during acute heat stress. Anim. Feed Sci. Technol..

[5] Kadono H, Besch E (1978). Telemetry measured body temperature of domestic fowl at various ambient temperatures. Poult. Sci..

[6] Yahav S (2009). Alleviating heat stress in domestic fowl: Different strategies. Worlds Poult. Sci. J..

[7] Al Wakeel RA, Shukry M, Abdel Azeez A, Mahmoud S, Saad MF (2017). Alleviation by gamma amino butyric acid supplementation of chronic heat stress-induced degenerative changes in jejunum in commercial broiler chickens. Stress.

[8] El-Naggar K, El-Kassas S, Abdo SE, Kirrella AAK, Al Wakeel RA (2019). Role of gamma-aminobutyric acid in regulating feed intake in commercial broilers reared under normal and heat stress conditions. J. Therm. Biol..

[9] Ncho CM, Jeong C, Gupta V, Goel A (2021). The effect of gamma-aminobutyric acid supplementation on growth performances, immune responses, and blood parameters of chickens reared under stressful environment: A meta-analysis. Environ. Sci. Pollut. Res..

[10] Dai SF (2011). Effects of dietary glutamine and gamma-aminobutyric acid on performance, carcass characteristics and serum parameters in broilers under circular heat stress. Anim. Feed Sci. Technol..

[11] Dalvi A, Rodgers R (2001). Anxiolytic effects of valproate and diazepam in mice are differentially sensitive to picrotoxin antagonism. Pharmacol. Biochem. Behav..

[12] Shekhar A (2006). Angiotensin-II is a putative neurotransmitter in lactate-induced panic-like responses in rats with disruption of GABAergic inhibition in the dorsomedial hypothalamus. J. Neurosci..

[13] Sherif F, Oreland L (1995). Effect of the GABA-transaminase inhibitor vigabatrin on exploratory behaviour in socially isolated rats. Behav. Brain Res..

[14] Ncho CM, Goel A, Jeong CM, Youssouf M, Choi YH (2021). In ovo injection of gaba can help body weight gain at hatch, increase chick weight to egg weight ratio, and improve broiler heat resistance. Animals.

[15] Ncho C-M, Goel A, Jeong C-M, Gupta V, Choi Y-H (2021). Effects of in ovo feeding of γ-aminobutyric acid on growth performances, plasma metabolites, and antioxidant status in broilers exposed to cyclic heat stress. Sustainability.

[16] Ncho CM, Goel A, Gupta V, Jeong C-M, Choi Y-H (2022). Embryonic manipulations modulate differential expressions of heat shock protein, fatty acid metabolism, and antioxidant-related genes in the liver of heat-stressed broilers. PLoS ONE.

[17] Ncho CM, Goel A, Gupta V, Jeong CM, Choi YH (2022). Effect of in ovo feeding of γ-aminobutyric acid combined with embryonic thermal manipulation on hatchability, growth, and hepatic gene expression in broilers. Anim. Biosci..

[18] Piestun Y, Halevy O, Yahav S (2009). Thermal manipulations of broiler embryos—The effect on thermoregulation and development during embryogenesis. Poult. Sci..

[19] Al-Zghoul MB, Mohammad Saleh KM (2020). Effects of thermal manipulation of eggs on the response of jejunal mucosae to posthatch chronic heat stress in broiler chickens. Poult. Sci..

[20] Saleh KM, Tarkhan AH, Al-Zghoul MB (2020). Embryonic thermal manipulation affects the antioxidant response to post-hatch thermal exposure in broiler chickens. Animals.

[21] Gouda A, Amer SA, Gabr S, Tolba SA (2020). Effect of dietary supplemental ascorbic acid and folic acid on the growth performance, redox status, and immune status of broiler chickens under heat stress. Trop. Anim. Health Prod..

[22] Harsini SG, Habibiyan M, Moeini MM, Abdolmohammadi AR (2012). Effects of dietary selenium, vitamin E, and their combination on growth, serum metabolites, and antioxidant defense system in skeletal muscle of broilers under heat stress. Biol. Trace Elem. Res..

[23] Lara LJ, Rostagno MH (2013). Impact of heat stress on poultry production. Animals.

[24] Al-Rukibat R, Al-Zghoul M, Hananeh W, Al-Natour M, Abu-Basha E (2017). Thermal manipulation during late embryogenesis: Effect on body weight and temperature, thyroid hormones, and differential white blood cell counts in broiler chickens. Poult. Sci..

[25] Al-Zghoul MB, Sukker H, Ababneh MM (2019). Effect of thermal manipulation of broilers embryos on the response to heat-induced oxidative stress. Poult. Sci..

[26] Collin A (2007). Effects of thermal manipulation during early and late embryogenesis on thermotolerance and breast muscle characteristics in broiler chickens. Poult. Sci..

[27] Lin H, Jiao HC, Buyse J, Decuypere E (2006). Strategies for preventing heat stress in poultry. Worlds Poult. Sci. J..

[28] Yahav S (1997). Induction of thermotolerance in chickens by temperature conditioning: Heat shock protein expression. Ann. N. Y. Acad. Sci..

[29] Chen XY, Wei PP, Xu SY, Geng ZY, Jiang RS (2013). Rectal temperature as an indicator for heat tolerance in chickens. Anim. Sci. J..

[30] Xie J (2014). Differential expression of heat shock transcription factors and heat shock proteins after acute and chronic heat stress in laying chickens (*Gallus gallus*). PLoS ONE.

[31] Lin H, Buyse J, Du R, Gu X, Zhang Z (2004). Response of rectal temperature of broiler chickens to thermal environment factors. Arch. fur Geflugelkd..

[32] Davis DM (2009). Nesting ecology and reproductive success of Lesser Prairie-Chickens in shinnery oak-dominated rangelands. Wilson J. Ornithol..

[33] Ouchi Y, Chowdhury VS, Cockrem JF, Bungo T (2021). Effects of thermal conditioning on changes in hepatic and muscular tissue associated with reduced heat production and body temperature in young chickens. Front. Vet. Sci..

[34] De Basilio V, Vilarino M, Yahav S, Picard M (2001). Early age thermal conditioning and a dual feeding program for male broilers challenged by heat stress. Poult. Sci..

[35] Piestun Y (2011). Thermal manipulations during broiler embryogenesis improves post-hatch performance under hot conditions. J. Therm. Biol..

[36] Maeda E, Kimura S, Yamada M, Tashiro M, Ohashi T (2017). Enhanced gap junction intercellular communication inhibits catabolic and pro-inflammatory responses in tenocytes against heat stress. J. Cell Commun. Signal..

[37] Shim K, Hwang K, Son M, Park GH (2006). Lipid metabolism and peroxidation in broiler chicks under chronic heat stress. Asian-Australas. J. Anim. Sci..

[38] Chowdhury VS, Tomonaga S, Nishimura S, Tabata S, Furuse M (2012). Physiological and behavioral responses of young chicks to high ambient temperature. J. Poult. Sci..

[39] Yahav S, Rath RS, Shinder D (2004). The effect of thermal manipulations during embryogenesis of broiler chicks (*Gallus domesticus*) on hatchability, body weight and thermoregulation after hatch. J. Therm. Biol..

[40] Ncho CM, Gupta V, Goel A (2021). Effect of thermal conditioning on growth performance and thermotolerance in broilers: A systematic review and meta-analysis. J. Therm. Biol..

[41] Jenkins SP (2005). Survival Analysis.

[42] Al-Zghoul MB, Saleh KM, Ababneh MMK (2019). Effects of pre-hatch thermal manipulation and post-hatch acute heat stress on the mRNA expression of interleukin-6 and genes involved in its induction pathways in 2 broiler chicken breeds. Poult. Sci..

[43] Tona K (2008). Effects of heat conditioning at d 16 to 18 of incubation or during early broiler rearing on embryo physiology, post-hatch growth performance and heat tolerance. Arch. für Geflügelkd. Sonderheft.

[44] Zaboli GR (2017). Thermal manipulation during pre and post-hatch on thermotolerance of male broiler chickens exposed to chronic heat stress. Poult. Sci..

[45] Ciampi A, Thiffault J, Nakache J-P, Asselain B (1986). Stratification by stepwise regression, correspondence analysis and recursive partition: A comparison of three methods of analysis for survival data with covariates. Comput. Stat. Data Anal..

[46] Percie du Sert N (2020). The ARRIVE guidelines 2.0: Updated guidelines for reporting animal research. PLoS Biol..

[47] Goel A, Ncho CM, Jeong CM, Choi YH (2021). Embryonic thermal manipulation and in ovo gamma-aminobutyric acid supplementation regulating the chick weight and stress-related genes at hatch. Front. Vet. Sci..

[48] Aviagen. *Aviagen Resource Center*, Vol. 0419-AVNAA-042 (2019).

[49] Goel A (2021). Dietary supplementation of shredded, steam-exploded pine particles decreases pathogenic microbes in the cecum of acute heat-stressed broilers. Animals.

[50] Purswell JL (2012). IX International Livestock Environment Symposium (ILES IX).

[51] Gál L, Oravec M, Kiššová M, Gemeiner P, Čeppan M (2020). Forensic discrimination of black laser prints by a combination of chemometric methods and μ-ATR-FTIR spectroscopy. Chem. Pap..

