# Supplementary material for: Impact of embryonic manipulations on core body temperature dynamics and survival in broilers exposed to cyclic heat stress
Source: Sci Rep. 2022 Sep 6;12:15110. doi: 10.1038/s41598-022-19063-1 (PMC9448727; doi:10.1038/s41598-022-19063-1)
Supplement: Supplementary file 1 — Supplementary Information. [file 41598_2022_19063_MOESM1_ESM.docx]

Supplementary information

**Impact of embryonic manipulations on core body temperature dynamics and survival in broilers exposed to cyclic heat stress**

Chris Major Ncho^1,2^, Akshat Goel ^1,3^, Vaishali Gupta^2^, Chae-Mi Jeong^1,2^, and Yang-Ho Choi^1,2,3^*

^1^ Department of Animal Science, Gyeongsang National University, Jinju 52828, Republic of Korea;

^2^ Division of Applied Life Sciences (BK21 Plus Program), Gyeongsang National University, Jinju 52828, Republic of Korea;

^3^ Institute of Agriculture and Life Sciences, Gyeongsang National University, Jinju 52828, Republic of Korea;

* Corresponding author; email: yhchoi@gnu.ac.kr

Supplementary Table 1: Descriptive statistics of the variables considered in the Cox proportional-hazards model

| Variable | N | Mean | Median | SE | Max | Min |
| --- | --- | --- | --- | --- | --- | --- |
| Time (min) | 117 | 330.7 | 358 | 4.9 | 358 | 83 |
| Temperature (°C) | 117 | 24.1 | 22.6 | 0.31 | 32.6 | 22.6 |
| Relative Humidity (%) | 117 | 61.4 | 65 | 0.8 | 65 | 21 |
| THI (°C) | 117 | 29.8 | 28.9 | 0.19 | 35.5 | 25.8 |
| Bodyweight (g) | 117 | 1468.6 | 1485 | 19.5 | 1935 | 940 |

Abbreviation: THI, Temperature-humidity index.

Supplementary Table 2: Average ambient temperature (T), relative humidity (RH), and temperature-humidity index (THI) variations in both thermoneutral and heat stress conditions during the trial

| Time (h) |  | 0 | 0.5 | 1 | 2 | 3 | 4 | 5 |
| --- | --- | --- | --- | --- | --- | --- | --- | --- |
| Thermoneutral | T(°C) | 22.5 | 22.5 | 22.5 | 22.7 | 22.9 | 22.5 | 22.6 |
|  | RH (%) | 50.9 | 52.7 | 51.6 | 50.1 | 51.7 | 51.0 | 50.9 |
|  | THI (°C) | 26.7 | 27.0 | 26.8 | 26.8 | 27.2 | 26.8 | 26.9 |
| Heat stress | T(°C) | 22.3 | 31.5 | 32.0 | 31.9 | 32.1 | 32.1 | 22.1 |
|  | RH (%) | 52.6 | 45.9 | 44.4 | 45.1 | 44.9 | 42.9 | 52.6 |
|  | THI (°C) | 26.9 | 33.6 | 33.9 | 33.9 | 34.0 | 33.7 | 26.7 |

Values indicate the mean of 7 days. From days 29 to 35, in the HS room, the environmental temperature was increased from 22 ± 1 °C to 32 ± 1 °C over 30 minutes, and this temperature was maintained for the next 4 hours before returning to 22 ± 1 °C over 30 minutes.. Abbreviation: THI, Temperature-humidity index.


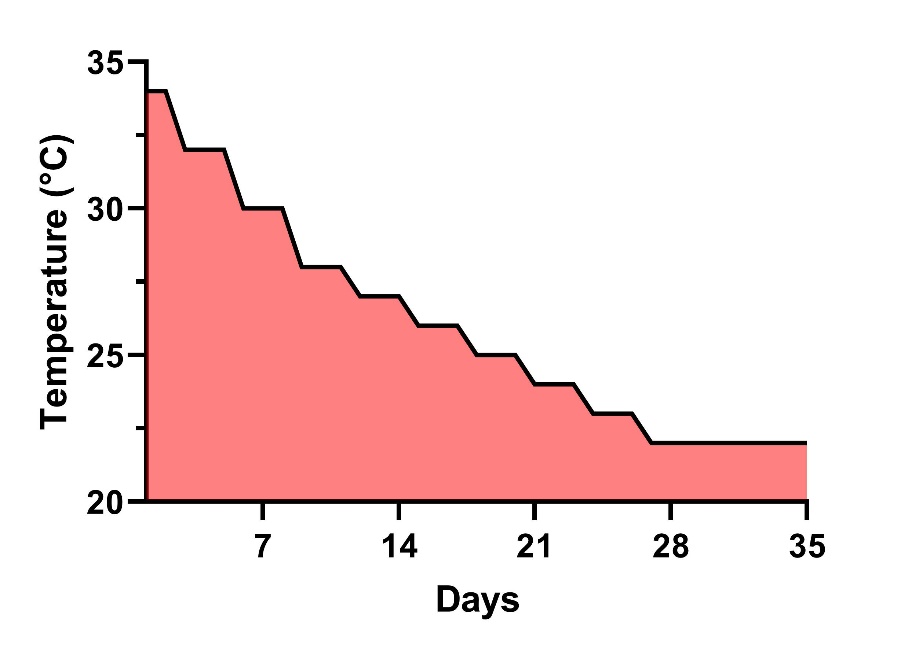


Supplementary Fig 1: Temperature evolution in the thermoneutral environment during the trial. After hatching, one-day-old broilers chicks were raised in battery brooders under a thermally controlled environment at 34 ± 1 °C and 50% RH, and then temperature was gradually decreased by 2 or 1 °C every three days to reached the recommended 22 ± 1 °C on day 28.
